# SOCIAL DISCONNECTEDNESS, PERCEIVED LONELINESS, AND COGNITIVE FUNCTIONING: THE ROLE OF NEIGHBORHOOD ENVIRONMENT

**DOI:** 10.1093/geroni/igae098.1565

**Published:** 2024-12-31

**Authors:** Fengyan Tang, Yanping Jiang

**Affiliations:** University of Pittsburgh, Wexford, Pennsylvania, United States; Rutgers, The State University of New Jersey, New Brunswick, New Jersey, United States

## Abstract

Social disconnectedness and loneliness pose significant challenges for older Chinese immigrants. This study aimed to investigate the association of social disconnectedness and loneliness with cognitive functioning and examine the moderation role of neighborhood contexts. This longitudinal analysis examined a sample of individuals aged 60 years and older from the Population Study of Chinese Elderly in Chicago (N=2,044). Global cognition was assessed using the averaged z-scores of cognitive performance tests. Social disconnectedness was constructed using five indicators about structural aspects of social relationships. Loneliness was assessed with the R-UCLA loneliness scale. Neighborhood socioeconomic status (NSES) and neighborhood segregation index (NSI) were constructed using 2010-2014 American Community Survey data at the census tract level. Individual perceptions about neighborhood environments were used to construct neighborhood cohesion index (NCI) and neighborhood disorder index (NDI). Latent growth curve models showed that more loneliness was associated with a higher level of initial cognitive functioning (B= 0.030, p<.01), but also with a faster decline rate over time (B= -0.007, p<.01) after adjusting for covariates. High NSES and less neighborhood segregation buffered the negative effects of loneliness on cognitive decline, respectively. High NDI amplified the positive relationship between loneliness and initial functioning, but accelerated the rate of cognitive decline associated with loneliness. The study revealed that perceived loneliness, but not social disconnectedness, is a risk factor for cognitive decline among older Chinese immigrants. Living in a neighborhood with low socioeconomic status, more segregation, and high disorder elevated the detrimental effect of loneliness on long-term cognitive decline.

